# Effectiveness of the Optimal Pulse Technology in a Novel Technique for Treating Nasal Blackheads in Asians: A Clinical Study

**DOI:** 10.1111/jocd.70493

**Published:** 2025-10-02

**Authors:** Qingmei Jin, Richeng Dong, Jiahui Zhi, Weilu Xu, Zhehu Jin, Chenglong Jin

**Affiliations:** ^1^ Department of Dermatology Yanbian University Hospital Yanji China; ^2^ Department of Dermatology Suzhou Mylike Cosmetic Hospital Suzhou China

**Keywords:** advanced optimal pulse technology, nasal blackhead, pore enlargement, treatment

## Abstract

**Background:**

Nasal blackheads, caused by the obstruction of hair follicles by sebum and exfoliated dead cells, represent a cutaneous issue that impairs facial aesthetics. Current therapeutic outcomes for this problem remain suboptimal.

**Aims:**

To evaluate the effectiveness and safety of a novel treatment mode (advanced OPT with medium energy, three pulses, long pulse width, AOPT‐MTL) for the treatment of nasal blackheads.

**Patients/Methods:**

A total of 42 patients with nasal blackheads were enrolled and treated using the AOPT‐MTL mode at intervals of 2–3 weeks for a total of 6 sessions. One month after the treatment was completed, the severity of nasal blackheads was assessed using the Enlarged Pore Classification Grade, and the treatment effectiveness rate was calculated. Patients were followed up for 6 months to evaluate recurrence, safety, and satisfaction.

**Results:**

After a course of treatment, the nasal blackheads of all patients were improved to varying degrees, and the treatment effectiveness rate reached as high as 95.2%. The Enlarged Pore Classification Grade score decreased from 5.40 ± 0.66 to 2.90 ± 0.61 (*p* < 0.05). No significant adverse reactions were observed during treatment. Recurrence was minimal during follow‐up, and patient satisfaction remained high.

**Conclusions:**

The AOPT‐MTL mode offers a simple, effective, and safe treatment option for nasal blackheads in Asians. With its demonstrated efficacy and good safety profile, it holds great promise for extensive clinical application.

## 1 Introduction

Blackheads are caused by the blockage of hair follicles with sebum, a substance secreted by the skin, and shed dead cells. They typically appear on the face—particularly on the nose—but may also develop on the back, chest, neck, arms, and shoulders. Blackheads are characterized by black‐tipped spots within visibly enlarged pores, and they can often be extracted in a worm‐like shape [1]. The nose is the most common and prominent site for blackhead formation. Among young women, nasal blackheads are considered a particularly concerning facial issue, as they can significantly affect physical appearance [2]. Consequently, there is a high demand for effective treatments targeting nasal blackheads.

**FIGURE 1:**
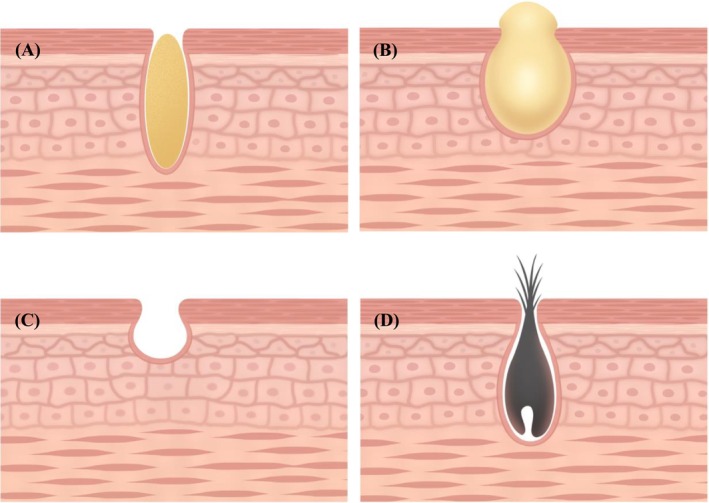
Schematic diagrams of the four types. (A) Lipid filament type: Lipid filament type is characterized by excessive sebum secretion combined with the accumulation of desquamated keratinocytes that are not effectively discharged, leading to pore obstruction. Upon exposure to air, the material undergoes oxidation and may appear brown or black. When manually expressed, it typically yields a yellowish—white, amorphous lipidic substance; (B) Keratin plug type: The keratin plug type can develop from the lipid filament type. In this case, the prolonged retention of sebum and keratinocytes within the pores results in the formation of a denser, more solidified mass. Over time, this material hardens into a relatively firm, light yellow, and translucent keratin plug. Due to its volume, this type is frequently associated with visible pore enlargement on the nasal skin; (C) Pore enlargement type: The pore enlargement type arises from persistent sebum overproduction, lack of timely dermatological intervention, or inappropriate treatment methods. These factors contribute to the progressive dilation of the nasal pores, which may occur independently or in conjunction with other blackhead types; (D) Vellus hair type: The vellus hair type represents a distinct subtype of nasal blackhead. In this form, sebum and exfoliated epithelial cells accumulate around vellus hairs within the pores, causing the hairs to become more prominent and visually noticeable.

The appearance of nasal blackheads is influenced by various factors, including gender, genetic predisposition, aging, ultraviolet (UV) radiation exposure, acne, and seborrheic dermatitis. Three primary mechanisms contribute to their formation: excessive sebum production, reduced skin elasticity around the pores, and increased hair follicle volume [3]. Nasal blackheads can be classified into four types: lipid filament type, keratin plug type, pore enlargement type, and vellus hair type (Figure 1: Schematic diagrams of the four types).

Current clinical treatment options for nasal blackheads include topical medications, chemical peels, ablative and non‐ablative lasers, radiofrequency microneedling, and localized botulinum toxin injections. However, these treatments are associated with several drawbacks, such as high recurrence rates, discomfort or pain, and extended recovery periods. Therefore, there is a pressing need for therapeutic approaches that are both minimally invasive and highly effective. Additionally, there is a notable lack of research on the classification of nasal blackheads and the clinical outcomes associated with their treatment.

Intense pulsed light (IPL) is a broad‐spectrum visible light therapy that can stimulate the reorganization and regeneration of collagen and elastic fibers in the skin, thereby enhancing skin elasticity and promoting structural repair. This results in skin rejuvenation. Furthermore, IPL has been shown to reduce sebum production, which helps alleviate issues related to oily skin. Advanced Optimal Pulse Technology (AOPT) represents the seventh‐generation enhancement of IPL, offering superior control over energy pulse delivery.

In this study, we employed the medium energy mode with three pulses and a long pulse width of AOPT to treat patients with nasal blackheads. We designated this novel treatment approach as “advanced OPT with medium energy, three pulses, and long pulse width (AOPT‐MTL)”. The objective of this article is to evaluate the effectiveness and safety of AOPT‐MTL in the treatment of nasal blackheads.

## 2 Materials and Methods

### 2.1 Ethics Statement

The study was approved by the relevant ethics committee and performed in accordance with the ethical standards stipulated in the 1964 Declaration of Helsinki. All patients provided written informed consent.

### 2.2 Participants

In this study, 42 patients with nasal blackheads who visited the clinic from June 2021 to December 2023 were selected. The average age of participants was 33.76 ± 7.98 years. Based on clinical classification, 16 patients presented with the lipid filament type, 13 with the keratin plug type, 8 with the pore enlargement type, and 5 with the vellus hair type. The severity of nasal blackheads was assessed using the Enlarged Pore Classification Grade developed by Wang et al. [4, 5], and only patients with a grade of 3 or above were included in the study. The specific evaluation method is shown in Table 1.

**TABLE 1:** Enlarged pore classification grade used for assessing nasal blackhead.

Grade	Description
Grade 1	Minimal visible pores
Grade 2	Slightly visible pores
Grade 3	Clearly visible pores
Grade 4	Clearly visible pores or pores filled with cornified cylindrical plugs smaller than pore size
Grade 5	Very clearly visible pores or pores filled with cornified cylindrical plugs of a size consistent with pores
Grade 6	Significantly enlarged pores or pores filled with cornified cylindrical plugs projecting from the pores, with a strawberry‐like appearance

The inclusion criteria were as follows: (1) Patients clinically diagnosed with nasal blackheads and with a severity rating > 3 points; (2) Patients aged ≥ 18 years old, regardless of gender; (3) Patients with Fitzpatrick skin types III to IV. The exclusion criteria were as follows: (1) Pregnant women, lactating women, or those planning to conceive; (2) Patients who had received facial treatment using other devices or participated in other clinical studies within 6 months prior to enrollment; (3) Patients with a history of exposure to or oral intake of photosensitizing drugs within one month prior to enrollment; (4) Patients with severe cardiorespiratory, liver, or kidney diseases; (5) Patients with cognitive impairment or mental disorders that could interfere with study participation; (6) Patients unable to return to the outpatient clinic on time or do not cooperate with the treatment plan.

### 2.3 Instrument

The ISEMECO skin imaging analyzer (Shanghai Meice Information Technology Company, China) and iPhone 11 Pro (Apple, California, USA) were used to evaluate the skin, and M22 (Lumenis Medical Company, USA) was used to provide AOPT‐MTL.

### 2.4 Procedures

Before treatment, each patient's nasal blackhead condition was evaluated and documented using either the ISEMECO skin imaging analyzer or a digital camera. Based on these assessments, patients were subsequently grouped for treatment. After evaluation, patients were instructed to cleanse their faces, and a thick layer (approximately 2–3 mm) of low‐temperature gel was applied to the treatment area. During the treatment, the patient shielded their eyes, and the operator wore goggles. The procedure began with the application of a 640 nm filter using three pulses. The pulse widths were set to 8.0–6.0–6.0 ms, with a pulse delay time of 40–40 ms. The energy density ranged from 16 to 20 J/cm^2^. Each treatment session with this filter was repeated three to four times. Following this, a 590 nm filter was selected, also using three pulses with the same pulse widths (8.0–6.0–6.0 ms) and pulse delay time (40–40 ms), and an energy density of 16 to 20 J/cm^2^. This step was also performed three to four times. The size of the light guide crystal used during the treatment is 15 mm × 35 mm. A cooling device was activated throughout the treatment to enhance patient comfort and reduce the risk of thermal injury. Specific parameters were adjusted based on each patient's skin color and Fitzpatrick skin type. The treatment endpoint was defined as the appearance of moderate erythema on the nasal skin, visible dilation of the follicular sebaceous gland openings, and the emergence of gray‐white, swollen blackhead keratin plugs on the skin surface. Routine post‐treatment care was administered. The interval between individual sessions was 2–3 weeks, with a total of six sessions constituting one complete course of treatment.

### 2.5 Outcomes

The Enlarged Pore Classification Grade was used to score and compare the severity of nasal blackheads in patients before treatment and one month after the completion of treatment. The improvement rate = (rating before treatment − rating after treatment)/rating before treatment × 100%. Based on the calculated improvement rate, treatment outcomes were categorized into three levels: mild improvement (≤ 30%), moderate improvement (31%–60%), and good improvement (> 60%). The effective treatment rate = (number of patients with moderate improvement + number of patients with good improvement)/total number of patients × 100%.

In addition to objective evaluation, patient satisfaction was assessed. Upon completion of the treatment, all patients completed a satisfaction questionnaire to evaluate their subjective perception of the treatment outcomes. The scale included four levels: very satisfied, satisfied, average, and dissatisfied. The satisfaction rate was calculated as follows: Satisfaction = (number of very satisfied cases + number of satisfied cases)/total number of cases × 100%.

### 2.6 Safety Evaluation

All patients were followed up continuously for 6 months to evaluate the safety and long‐term effects of the treatment. During the follow‐up period, any adverse reactions were documented, including pain intensity, erythema, edema, hyperpigmentation, hypopigmentation, scarring, and other skin‐related complications. In addition, a clinical reassessment of the nasal skin was conducted 6 months after the final treatment session to monitor for any signs of recurrence.

### 2.7 Statistical Analysis

Normally distributed data were expressed as mean ± standard deviation. The paired *t*‐test was used for within‐group comparisons. The Statistical Package for Social Science version 25.0 and Graphpad Prism 8.0 were employed for statistical analysis. A *p*‐value < 0.05 was deemed statistically significant.

## 3 Results

**FIGURE 2:**
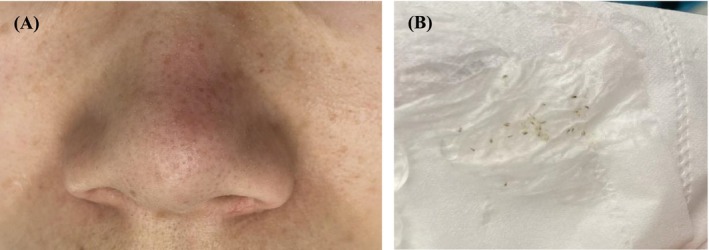
Endpoint reactions of the skin during treatment. (A) Immediately after the treatment, a moderate erythema reaction was observed on the nasal skin. The openings of the follicular sebaceous glands became visibly dilated, and the keratin plugs of the nasal blackheads changed in appearance, turning grayish‐white, swelling, and protruding from the skin surface. (B) Within the first 3 days after each treatment session, some of the keratin plugs detached spontaneously.

**FIGURE 3:**
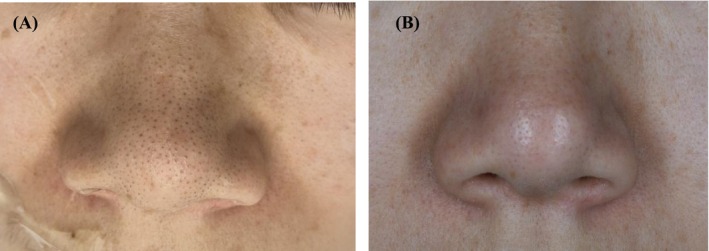
Pre‐therapy and posttreatment images of lipid filament type. (A) Before treatment; (B) After treatment.

**FIGURE 4:**
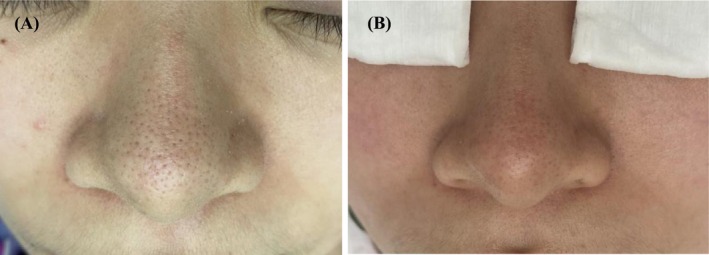
Pre‐therapy and posttreatment images of keratin plug type. (A) Before treatment; (B) After treatment.

**FIGURE 5:**
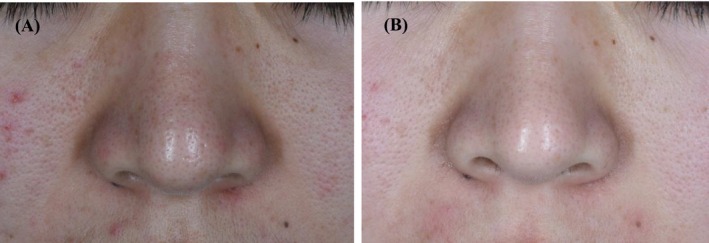
Pre‐therapy and posttreatment images of pore enlargement type. (A) Before treatment; (B) After treatment.

**FIGURE 6:**
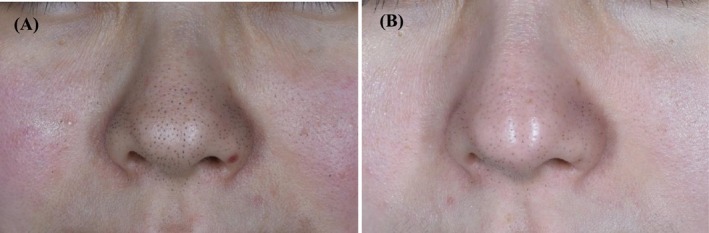
Pre‐therapy and posttreatment images of vellus hair type. (A) Before treatment; (B) After treatment.

All 42 patients completed the full course of treatment and follow‐up. The clinical characteristics of the patients with nasal blackheads are summarized in Table 2. Based on clinical photographic observations, a moderate erythema reaction was observed on the nasal skin immediately following treatment. The openings of the follicular sebaceous glands became visibly dilated, and the keratin plugs of the nasal blackheads changed in appearance, turning grayish‐white, swelling, and protruding from the skin surface (Figure 2A). Within the first 3 days after each treatment session, some of the keratin plugs detached spontaneously (Figure 2B). Upon completion of the full treatment course, varying degrees of improvement were observed across all four nasal blackhead types (Figures 3, 4, 5, 6).

**TABLE 2:** Clinical characteristics of 42 patients with nasal blackhead.

Variables	Number of patients (%)
Total cases	42
Age (y)	
Mean (range)	33.8 (19–49)
Duration (y)	
Mean (range)	9.2 (2–20)
Sex	
Male/female	11 (26.2)/31 (73.8)
Type of nasal blackhead	
Sebum increased type	16 (38.1)
Keratin plug type	13 (31.0)
Pore enlargement type	8 (19.0)
Vellus hair type	5 (11.9)

**FIGURE 7:**
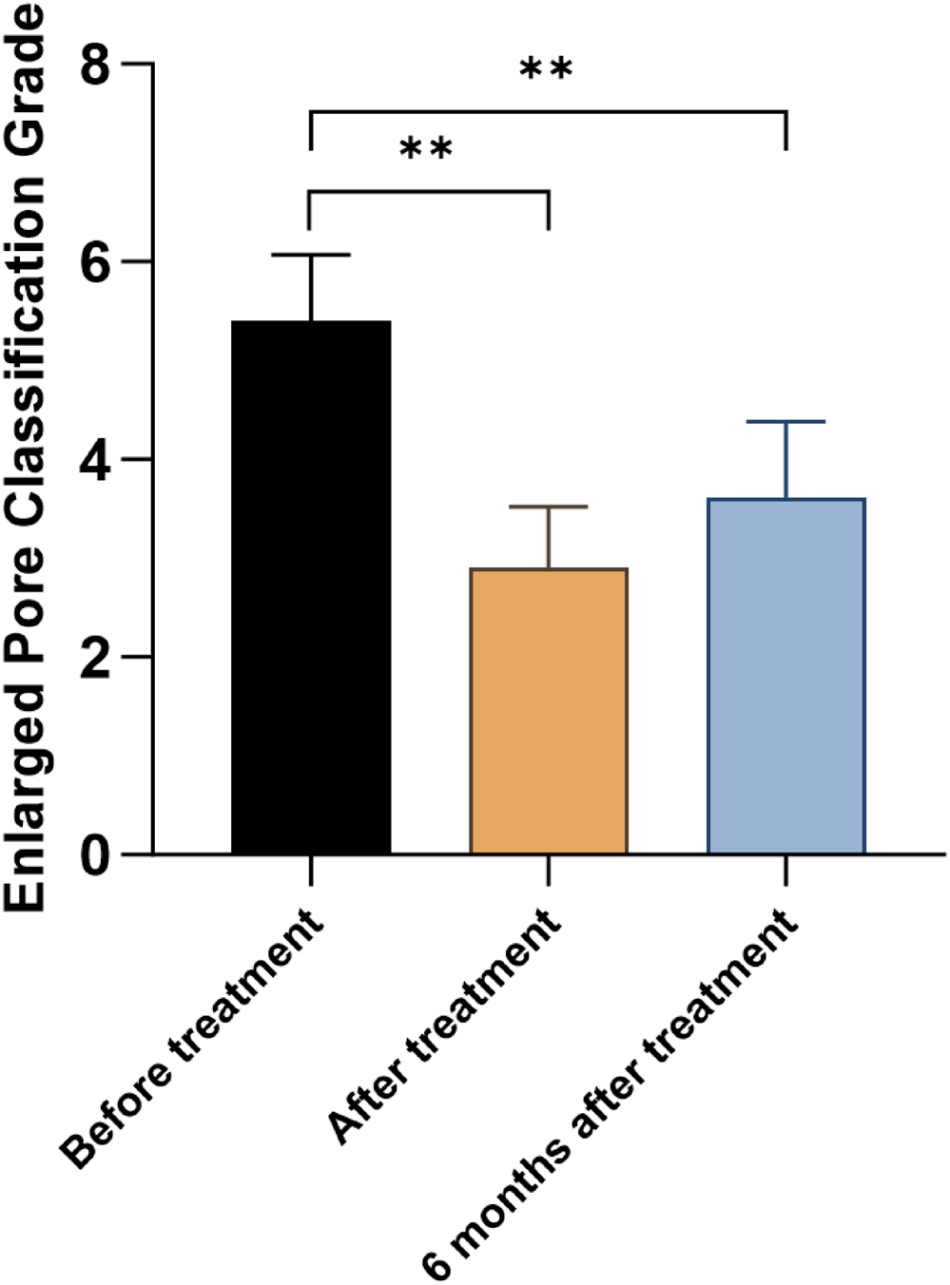
Comparison of the Enlarged Pore Classification Grade of all patients before treatment and after treatment. *p < 0.05 and **p < 0.01.

**FIGURE 8:**
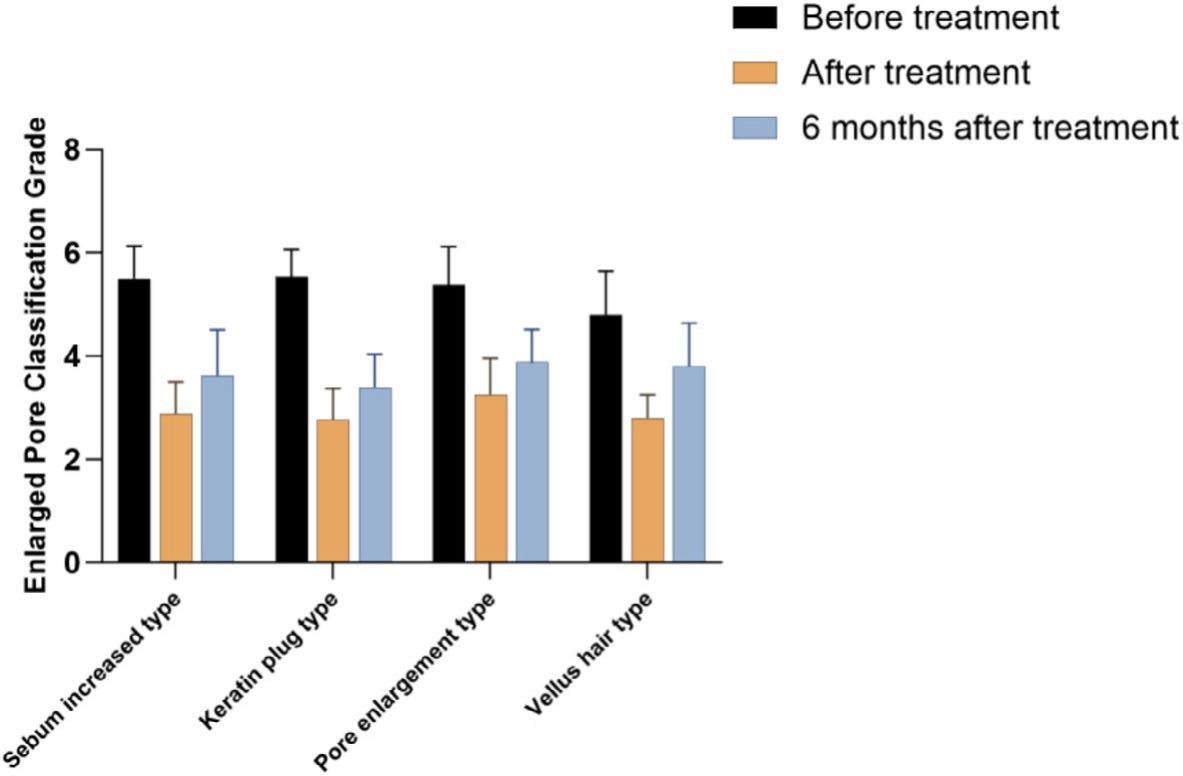
Improvement of the enhanced pore classification grade for each type of Nasal blackhead.

### 3.1 Analysis of Treatment Effectiveness

After the full course of treatment, the *Enlarged Pore Classification Grade* for all patients significantly decreased from 5.40 ± 0.66 to 2.90 ± 0.61, with the difference reaching statistical significance (*p* < 0.05). The overall treatment effective rate was 95.2%. The changes in classification scores and improvement rates before and after treatment for each of the four types of nasal blackheads are presented in Figures 7, 8, and Table 3, respectively. In all cases, the differences observed before and after treatment were statistically significant (*p* < 0.05).

**TABLE 3:** Overall improvement of nasal blackhead.

Improvement (%)	Cases (%)				
	Total	Sebum increased type	Keratin plug type	Pore enlargement type	Vellus hair type
≤ 30	2 (4.8)	0	0	1 (12.5)	1 (20.0)
31–60	35 (83.3)	14 (87.5)	10 (76.9)	7 (87.5)	4 (80.0)
> 60	5 (11.9)	2 (12.5)	3 (23.1)	0	0

### 3.2 Safety and Patient Satisfaction

During the treatment process, patients only experienced mild pain, with a VAS score of 2.90 ± 1.09. Moderate erythema reactions appeared on the skin but subsided within 30 min, and no obvious edema was observed. During the follow‐up visits, no adverse effects such as hyperpigmentation, hypopigmentation, or scarring were reported. At 6 months post‐treatment, although there was a slight increase in the Enlarged Pore Classification Grade compared to immediately after treatment, the scores remained significantly improved relative to baseline values. The overall patient satisfaction rate was 90.48%, as summarized in Table 4.

**TABLE 4:** Subject satisfaction.

	Cases (%)				
	Total	Sebum increased type	Keratin plug type	Pore enlargement type	Vellus hair type
Extremely satisfied	20 (47.63)	6 (37.5)	6 (46.15)	4 (50)	4 (80)
Very satisfied	18 (42.86)	7 (43.75)	7 (53.85)	3 (37.5)	1 (20)
Slightly satisfied	4 (9.52)	3 (18.75)	0	1 (12.5)	0
Not satisfied	0	0	0	0	0

## 4 Discussion

Blackheads are primarily formed due to excessive sebum secretion, which leads to the obstruction of hair follicle openings. Upon prolonged exposure to air, the sebum undergoes oxidation and is further exacerbated by environmental contaminants such as dirt and dust [6]. On the face, blackheads are most commonly observed on the nose, an area with particularly active sebaceous glands. Given that the nose occupies a central position on the face, the presence of nasal blackheads can significantly affect facial aesthetics, thereby creating a high demand for effective clinical treatment. Anatomically, the nasal skin contains a high density of sebaceous glands, resulting in a greater volume of sebum secretion compared to other facial regions [7]. Prolonged accumulation of sebum in the pores can gradually increase pore diameter. With skin aging, there is a progressive decline, deformation, and rupture of dermal components such as elastic fibers and collagen fibers. This degradation contributes to skin laxity and impairs the ability of the pores to remain tight, eventually leading to the development of enlarged pores [8]. Histological studies have shown that enlarged pores may appear as empty, funnel‐shaped structures or keratinized cylindrical plugs, which correspond to comedones [9, 10]. Therefore, the formation of blackheads and the enlargement of pores on the nose are complementary processes.

At present, the main methods for treating nasal blackheads include topical medications, chemical peels, ablative or non‐ablative lasers, and radiofrequency microneedling [11]. Topical medications or chemical peels—such as those containing fruit acids—primarily function by dissolving excessive keratin and reducing sebum secretion. However, their therapeutic effects are often limited and associated with a high recurrence rate. Energy‐based treatments, such as lasers and radiofrequency microneedling, can inhibit sebaceous gland activity and stimulate collagen contraction and regeneration around the pores. Nonetheless, these procedures are often accompanied by considerable discomfort and extended downtime. Inadequate postoperative care may lead to complications such as infection, pigmentation, or scarring [3, 5]. In some cases, patients attempt to remove blackheads at home by squeezing the nasal area or using peel‐off masks. These improper techniques not only fail to resolve the underlying condition but may also aggravate it, resulting in skin infections, further pore dilation, and capillary damage. Therefore, there is a pressing need for a treatment approach that offers minimal discomfort, is non‐invasive, and requires no downtime. At present, there remains a scarcity of clinical studies specifically addressing nasal blackhead treatment. Effective management should simultaneously target multiple pathological factors, including inhibition of sebum production, facilitation of keratin plug removal, repair of damaged collagen, and stimulation of new collagen synthesis.

Intense pulsed light (IPL), also referred to as photorejuvenation, is a broad‐spectrum, non‐coherent visible light with wavelengths ranging from 400 to 1200 nm. The energy delivered by IPL at different wavelengths can be selectively absorbed by various chromophores within the skin, thereby achieving targeted therapeutic effects. IPL has been widely employed in dermatology, particularly for facial rejuvenation [12]. The thermal effects of IPL are primarily confined to the papillary and reticular dermis, where they stimulate fibroblast proliferation and activity, leading to an increase in type I and type III collagen content [13]. This dermal remodeling enhances epidermal thickness, reduces keratin accumulation, promotes the regeneration of rete ridges, and ultimately results in wrinkle reduction, improved skin elasticity, and decreased pore size. Additionally, IPL induces thermal damage to the sebaceous glands within hair follicles, thereby reducing sebum production [14]. Compared to traditional pulsed wave, AOPT eliminates the energy peak at the onset of the pulse and avoids the energy decay at the end, making the energy output more stable. This not only improves the treatment effectiveness but also reduces the incidence of adverse reactions, making the treatment safer [15].

In this study, we utilized the AOPT platform of the M22 instrument manufactured by Lumenis. Initially, a 640 nm filter was employed to stimulate the contraction and regeneration of dermal collagen fibers, thereby improving skin firmness. This wavelength penetrates deeply into the dermis while minimizing epidermal damage [16]. Additionally, the 640 nm light can reach and thermally impair deep‐seated sebaceous glands, promoting glandular atrophy and subsequently reducing sebum secretion—an effect that supports the long‐term resolution of blackheads. Subsequently, a 590 nm filter was applied. Following treatment with this filter, blackheads on the nasal skin appeared grayish‐white and visibly emerged on the surface. This phenomenon may be attributed to the preferential absorption of 590 nm light by oxidized keratin plugs, which then expand upon heating. The expansion loosens the adhesion between the keratin plugs and the follicular wall, facilitating their expulsion. The use of a triple‐pulse mode with a pulse duration of 8–6–6 ms in the long pulse width setting allows for sufficient thermal conduction to the entire hair follicle–sebaceous gland unit, promoting a controlled process of thermal damage and subsequent tissue repair. Moreover, the brief thermal stimulation induced by IPL activates the arrector pili muscles, which contract and exert pressure on the sebaceous glands, further assisting in the extrusion of keratin plugs from the pores. Therefore, when applied to facial pores—including nasal blackheads—AOPT exerts a multi‐targeted therapeutic effect on collagen, sebaceous glands, keratin plugs, and the arrector pili muscles. This integrated mechanism of action shortens the treatment cycle and reduces recurrence. Following treatment with the AOPT‐MTL mode, blackheads thermally stimulated during the procedure continued to be metabolized and shed naturally within 3 days. This avoids the mechanical trauma and potential complications associated with methods such as manual extraction or vacuum suction.

To facilitate the observation and statistical analysis of treatment outcomes, nasal blackheads were categorized into four distinct types: lipid filament type, keratin plug type, pore enlargement type, and vellus hair type. As illustrated in the schematic diagram (Figure 1), the lipid filament type generally represents the initial stage of nasal blackhead formation. In this type, excessive sebum secretion, combined with the accumulation of desquamated keratinocytes that are not effectively discharged, leads to pore obstruction. Upon exposure to air, the material undergoes oxidation and may appear brown or black. When manually expressed, it typically yields a yellowish‐white, amorphous lipidic substance. The keratin plug type can develop from the lipid filament type. In this case, the prolonged retention of sebum and keratinocytes within the pores results in the formation of a denser, more solidified mass. Over time, this material hardens into a relatively firm, light yellow, and translucent keratin plug. Due to its volume, this type is frequently associated with visible pore enlargement on the nasal skin. The pore enlargement type arises from persistent sebum overproduction, lack of timely dermatological intervention, or inappropriate treatment methods. These factors contribute to the progressive dilation of the nasal pores, which may occur independently or in conjunction with other blackhead types. The vellus hair type represents a distinct subtype of nasal blackhead. In this form, sebum and exfoliated epithelial cells accumulate around vellus hairs within the pores, causing the hairs to become more prominent and visually noticeable.

A total of 42 patients with four types of nasal blackheads were included in this study to evaluate the therapeutic effect of the AOPT‐MTL mode on nasal blackheads. The treatment outcomes for each blackhead type were analyzed individually. The results indicated a progressive improvement in therapeutic effect with an increasing number of treatment sessions. One month after the completion of treatment, all patients showed varying degrees of improvement in the severity of nasal blackheads, with an overall improvement rate of 95.2%. When examining the treatment efficacy across the four subtypes, the keratin plug type exhibited the most significant reduction in the Enlarged Pore Classification Grade score. This may be attributed to the observation that, once the keratin plug is expelled from the pore, it does not readily reform within a short period. Consequently, this type also demonstrated the lowest classification score at the 6‐month follow‐up. This was followed by the lipid filament type and the pore enlargement type. Although the reduction in the Enlarged Pore Classification Grade score for the vellus hair type was less pronounced compared to the other subtypes, this group reported the highest patient satisfaction. These findings may serve as a valuable reference for clinicians in selecting appropriate treatment indications based on blackhead type. At the 6‐month follow‐up, although there was a slight increase in the Enlarged Pore Classification Grade score compared to the 1‐month post‐treatment assessment, the scores remained significantly improved relative to baseline. Accordingly, we recommend that patients undergo intermittent maintenance therapy over the long term. Additionally, patients should be advised to maintain consistent skincare routines to minimize excessive sebum production, thereby helping to sustain therapeutic benefits and reduce recurrence rates.

We acknowledge certain limitations in this study, including the relatively small sample size, the absence of a control group, and the lack of longer‐term follow‐up. Future studies with expanded cohorts and extended observation periods are planned to further address these limitations.

## 5 Conclusion

The treatment of nasal blackheads in Asians using the AOPT‐MTL mode is a simple, efficient, and safe treatment method and has excellent prospects for clinical application.

## Author Contributions

Q.J. and R.D. designed the study, analyzed the data, and wrote the manuscript. J.Z. and W.X. contributed to the collection of clinical data. Q.J., R.D., Z.J., and C.J. reviewed and revised the manuscript. Z.J. and C.J. conceived of and supervised the study. All authors have read and approved the final manuscript.

## Ethics Statement

The study was performed in accordance with the ethical standards stipulated in the 1964 Declaration of Helsinki and was approved by the Medical Ethics and Human Research Committee of Suzhou Mylike Cosmetic Hospital of China. Ethical No: 2021003.

## Consent

All patients provided written informed consent. A total of 42 patients with nasal blackheads were recruited from Suzhou Mylike Cosmetic Hospital.

## Conflicts of Interest

The authors declare no conflicts of interest.

## Data Availability

The data that support the findings of this study are available from the corresponding author upon reasonable request.
